# Effectiveness and Safety of Lacosamide, A Third-generation Anti-seizure Medication, for Poststroke Seizure and Epilepsy: A Literature Review

**DOI:** 10.2174/1570159X21666230616114255

**Published:** 2023-08-15

**Authors:** Yu-Shiue Chen, Ming-Chi Lai, Tsang-Shan Chen, Yung-Hsin Tseng, Ya Jhen Li, Chin-Wei Huang

**Affiliations:** 1 Department of Neurology, National Cheng Kung University Hospital, College of Medicine, National Cheng Kung University, Tainan, Taiwan;; 2 Department of Pediatrics, Chi-Mei Medical Center, Tainan, Taiwan;; 3 Department of Neurology, Tainan Sin-Lau Hospital, Tainan, Taiwan;; 4 Department of Pharmacy, National Cheng Kung University Hospital, College of Medicine, National Cheng Kung University, Tainan, Taiwan;; 5 Kun-Yen Medical Library, National Cheng Kung University, Tainan, Taiwan

**Keywords:** Lacosamide, poststroke, seizure, epilepsy, anti-seizure medication, effectiveness, safety

## Abstract

Advances in stroke treatment have resulted in a dramatic reduction in stroke mortality. Nevertheless, poststroke seizures and epilepsy are issues of clinical importance affecting survivors. Additionally, stroke is the most common cause of epilepsy in older adults. Although numerous antiseizure medications exist, studies are needed to provide robust evidence of the efficacy and tolerability of these medicines for treating poststroke seizures and epilepsy. Crucially, the newer generations of antiseizure medications require testing. Lacosamide, a third-generation antiseizure medication approved for treating localization-related epilepsy, has a novel mechanism of selectively enhancing the slow inactivation of sodium channels. This literature review evaluated whether lacosamide is effective and safe for the treatment of poststroke seizures and epilepsy. This review critically analyzed studies published in major academic databases (Pubmed, Embase, and Cochrane Library) from inception through June 2022 regarding the interaction of lacosamide with poststroke seizures and epilepsy. We included clinical prospective, retrospective, and case studies on patients with poststroke seizure and epilepsy, lacosamide as a treatment for seizures, neuroprotection in animal models of seizures, and the safety of lacosamide when coadministering anticoagulants. Clinical studies revealed lacosamide to be an effective antiseizure medication with high efficacy and tolerability in patients with poststroke seizures and epilepsy. In animal models, lacosamide proved effective at seizure reduction and neuroprotection. Pharmacokinetic studies demonstrated the safety of lacosamide when coadministering conventional and new anticoagulants. The literature suggests that Lacosamide is a promising candidate antiseizure medication for patients with poststroke seizures and epilepsy.

## 1 INTRODUCTION

Advances in stroke treatment have resulted in a dramatic reduction in stroke mortality; however, the number of survivors living with morbidities has increased substantially. Seizures and epilepsy are common morbidities among survivors of stroke, and poststroke seizures and epilepsy (PSSE) have been identified as major clinical issues with both medical and psychosocial aspects [1-3]. Stroke is the most common cause of epilepsy in older adults (aged 65 and older) [4]. Among survivors of stroke, the incidence of the early seizure (occurring within the first 1-2 weeks of stroke) is between 2.4% and 6.4% and the risk of late seizure (occurring more than 14 days after stroke) is approximately 7-18% [4, 5]. PSSE accounts for 30-50% of new-onset seizures in older adults [4]. An epidemiological study in Rochester, Minnesota, USA, of 489 patients with first ischemic stroke and a mean of 6.5 years of follow-up revealed that 7.2% experienced new-onset seizures. Among them, 11.4% developed status epilepticus [6]. A systematic review and meta-analysis revealed that cortical involvement, severe stroke, hemorrhagic transformation, age < 65 years, large lesion, and atrial fibrillation were the major risk factors for poststroke early seizures, whereas cortical involvement, cerebral hemorrhage, and early seizure were associated with an increased risk of poststroke epilepsy [2, 4, 7, 8]. Among patients with stroke, the risk of subsequent unprovoked seizures after the first acute symptomatic seizure was 33.0%, and the rate of additional unprovoked seizures after the first one was 71.5% [9]. During the first year after ischemic stroke in the anterior circulation, 25.2% of patients had an epileptic seizure. During hospitalization, electroencephalography (EEG) revealed epileptiform activity (interictal or ictal) in 17.9% of patients, and 25.9% of epileptiform activity indicated electrographic seizures [10]. Functional outcomes of the index seizure worsened after the stroke, as reflected by a 0.4 mean increase on a modified Rankin scale [6]. Our previous study documented how a seizure at stroke presentation and during hospitalization worsens overall morbidity and mortality [2], suggesting the importance of seizure care as a component of short-term and long-term care of patients with ischemic stroke. Most patients with stroke require rehabilitation to regain functionality for daily living. PSSE can substantially impair patient quality of life and increase caregiver burden. Sixty percent of patients and nursing staff associated effective seizure control, including freedom from PSSE, with quality of life [10]. In the guidelines on managing PSSE issued by the European Stroke Organization, the administration of secondary antiseizure medications (ASMs) as prophylaxis was recommended because of the high incidence of seizure recurrence after the initial poststroke unprovoked seizure [11]. A large, nationwide, population-based study using the Taiwan National Health Insurance Research Database (NHIRD) assessed the efficacy of various ASMs for controlling poststroke epilepsy. Most patients (69%) were prescribed phenytoin to manage poststroke seizures, followed by valproate (20%), new ASMs (oxcarbazepine, vigabatrin, tiagabine, topiramate, gabapentin, levetiracetam, and pregabalin; 7%) and carbamazepine (4%). Among patients with late-onset poststroke epilepsy, valproate and new ASMs provided better seizure control than phenytoin, as demonstrated by fewer emergency department visits and lower risks of hospitalization [12]. Another large retrospective study using the NHIRD found that 86% of patients with poststroke epilepsy were prescribed conventional ASMs, with phenytoin accounting for 56% of prescriptions. Only 14% of patients were prescribed newer ASMs [13]. Phenytoin use was associated with a higher risk of death within 5 years [13]. A systematic review with network meta-analysis on randomized controlled trials of ASMs for the treatment of poststroke seizures found no significant differences in freedom from seizure between either lamotrigine or levetiracetam and carbamazepine-controlled release (CBZ-CR). Levetiracetam and lamotrigine were better tolerated than CBZ-CR, and levetiracetam was associated with more adverse events than lamotrigine in this study. Further studies are needed to provide robust evidence on the efficacy and tolerability of ASMs for treating poststroke epilepsy [14]. Lacosamide (LCM), a third-generation ASM with a distinct novel mechanism of selectively enhancing the slow inactivation of sodium channels, has demonstrated potential as a treatment for various epileptic disorders [15-17]. We have also demonstrated the frequency-dependent inhibition of voltage-gated sodium channels by LCM [18]. The efficacy and safety of LCM for treating localization-related epilepsy have been established in various clinical and experimental studies, including our own [16, 19-21]. LCM has demonstrated a fast onset of antiseizure activity and it effectively reduces focal seizures at doses of 200-400 mg/day as an adjunct therapy in localization-related epilepsy [17, 21, 22]. The outstanding clinical performance of LCM has raised its profile as a potential candidate for treating patients with poststroke epilepsy. Accordingly, this literature review evaluates whether LCM has high efficacy and safety for the treatment of patients with PSSE.

## 2 MATERIALS AND METHODS

### 2.1 Search Strategy

We conducted a literature review to identify studies reporting the efficacy and safety of LCM for treating PSSE. Our search included both human and animal studies, did not impose article type or year restrictions, and collected original articles, case reports, clinical trials, meta-analyses, reviews, and systematic reviews. We searched three academic databases: PubMed, Embase, and Cochrane Library. The search period lasted until June 10, 2022, and the keywords used were “seizure/epilepsy”, “cerebral vascular accident/post stroke”, and “Lacosamide/Vimpat”. Controlled vocabulary (*e.g*., MeSH or Emtree search were employed) included Boolean logic combinations of keywords. Two researchers collected all papers that met the search criteria and then included several additional papers based on reference lists. The initial screening of the search results involved the inspection of article titles and abstracts. The researchers then screened the full text of all articles considered for inclusion. Articles were excluded if, upon inspection, they were found to not contain information regarding LCM and PSSE. The search process is outlined in Fig. (**1**).

### 2.2 Eligibility Criteria of Clinical Studies

All included clinical studies discussed LCM as a treatment for cerebral vascular accident/post stroke and seizure/epilepsy and included discussions of efficacy (seizure frequency, percentage of seizure freedom) and safety (incidence of adverse effects). In accordance with our study objective, the selection and inclusion criteria for clinical studies were as follows:

1) Study focused on LCM therapy for PSSE.

2) Treatment involved either LCM alone (monotherapy) or LCM combined with one or more other ASMs (polytherapy).

3) The effectiveness and safety of LCM were assessed in the paper (including the seizure frequency, percentage of seizure freedom, and adverse events).

## 3 RESULTS AND DISCUSSION

### 3.1 Search Results

On the basis of the screening criteria, 257 studies were selected, and 239 records remained after screening for duplicates. The titles and abstracts of the remaining studies were examined carefully, and 208 irrelevant articles were removed, as were 22 pieces of gray literature and conference posters. Finally, nine articles (seven human studies and two animal studies) were selected for analysis. An outline of the search process is presented in Fig. (**1**).

### 3.2 Human Studies of LCM for PSSE

Seven human studies were included in the literature review; two were prospective studies, three were retrospective observational studies, and two were case reports.

#### 3.2.1 Prospective Studies

Rosenow *et al.* [23] reported on LCM therapy for 174 patients with cerebrovascular epilepsy etiology (CVEE). Their report, an exploratory post hoc analyses, detailed the study results of three standard clinical trials. The first study was a randomized, double-blind, noninferiority, initial monotherapy trial of LCM (initial 100 mg/day, first target dose 200 mg/day, later flexible up-titration of 400/600 mg/day) *versus* CBZ-CR (initial 200 mg/day, first target dose 400 mg/day, later flexible up-titration to maximal 800/1200 mg/day). The vascular etiology was “cerebrovascular accident”. This study revealed a higher proportion of seizure freedom in the LCM group (LCM 81.5% *versus* CBZ-CR 58.5% at 6 months, and LCM 66.7% *versus* CBZ-CR 50.0% at 12 months). As for the treatment-emergent adverse events (TEAEs), 74.1% of patients on LCM *versus* 79.4% on CBZ-CR reported headaches and dizziness as the main TEAE. The rates of discontinuation due to TEAEs were 20.6% in the CBZ-CR group and 11.1% in the LCM group. In the second study (a randomized, double-blind, historical-controlled, conversion to LCM monotherapy trial of 200-400 mg/day) and the third study (an observational study of adjunctive LCM added to one ASM at physician’s discretion), the etiology category was “vascular causes” or “cerebrovascular etiology”. The percentages of 50% responders, 75% responders, and seizure freedom were 56.7%, 40%, 20% and 80%, 74.7%, and 56% for the second and third studies, respectively. The rates of discontinuation due to TEAEs were 16.7% and 9.6% in the two studies, and the most common TEAEs were dizziness (26.7%) and fatigue (14.5%). These studies indicated that LCM was relatively well tolerated and effective in patients with CVEE, both as monotherapy and as add-on therapy (Table **1**). As the three studies were exploratory post hoc analyses, not primary studies recruiting patients with post-stroke epilepsy, the inclusion criteria as CVEE might be relatively heterogeneous, in terms of the etiology of vascular events. In addition, the three studies differed in the design of the trial, age of patients, starting and maintenance dose, duration and patient eligibility. Nevertheless, the studies evaluated the efficacy and adverse effects carefully, and the results supported their hypothesis. Sharma *et al.* [24] reported on 52 patients with refractory status epilepticus, with ischemic stroke as the most common identifiable etiology (remote or acute, 16.7%), receiving LCM after the failure of second-line medication treatment. LCM could rapidly reach maximum plasma concentration within 1-4 h, and the number of patients who responded to LCM increased from 17.3% at 4 h to 78.8% in 48 h. This study also revealed no significant differences in renal function after LCM administration on days 1 and 7. Accordingly, the researchers concluded that LCM was an ideal ASM for patients with critical illnesses. Although ischemic stroke was the most common identifiable etiology in this study, it is not uncommon to encounter patients with refractory status epilepticus who have other comorbidities as potential etiology, such as infection or other systemic insults. The medications prior to LCM were not systematically analyzed, in terms of combination with LCM. The specific monitoring of hepatic and renal function during LCM therapy was informative in clinical practice. Accordingly, the researchers concluded that LCM was an ideal ASM for patients with critical illnesses. The above prospective studies indicated that LCM was well tolerated and effective in patients with CVEE, including patients with refractory status epilepticus.

#### 3.2.2 Retrospective Observational Studies

Belcastro *et al.* [25] reported on 16 older adult patients with poststroke nonconvulsive status epilepticus (NCSE). Patients were administered an initial 400 mg loading dose of intravenous (IV) LCM, followed by a mean maintenance dose of 400 mg/day. LCM was effective in half of the participants, in whom epileptic activity disappeared (87.5%) or decreased (12.5%) within 1 h, with no relapse over the following 24 h and no adverse events. Most of the patients in this study were acute seizures following stroke. The patients underwent video-EEG monitoring in a relatively short duration, although patients with NCSE typically need a longer duration of monitoring and the duration of status epilepticus prior to LCM therapy could be an important variable. Nevertheless, this pilot study showed the safety and efficacy profile of LCM in patients with poststroke NCSE. Mnatsakanyan *et al.* [26] reported on 10 patients with refractory NCSE, including one patient with left hemispheric chronic infarct and NCSE. All patients were given IV LCM after the standard treatment for status epilepticus failed to control seizures within 1-2 days. The median loading dose was a 200-300 mg infusion within 30 minutes, and the maintenance dose was 100-200 mg per 12 h. Seven of the 10 patients reached the resolution of NCSE under this treatment. An EEG of the patient with left hemispheric chronic infarct revealed localization of ictal patterns over the left posterior quadrant. This patient was administered baseline phenytoin, levetiracetam, and valproate before the addition of LCM. The seizure was improved by levetiracetam, valproate, and LCM at the time of discharge. In summary, this study indicated that IV LCM can be an efficacious and safe adjunctive agent for the treatment of refractory NCSE, but the generalizability of these findings was limited by the small sample size and the number of participants with a stroke etiology. Moreover, patients with poststroke epileptic seizures who need multiple (three) ASMs are relatively less common. Guilhoto *et al.* [22] reported the pediatric experiences of 16 patients, including two patients with stroke and drug-resistant focal epilepsy, who received adjunctive LCM therapy (dosed at 4.7 mg/kg daily; mean dose of 275 mg/day). Patients received a median of two ASMs. Generally, this study demonstrated a beneficial response to LCM, with a median seizure reduction of 39.6% (range 26-40%) and without severe adverse events. The two patients with infarct demonstrated no marked reduction in their high baseline seizure frequency (60 and 90 per month) after LCM use for 1 month and 8 months, respectively. Of these two patients, one reported nausea, diplopia, blurred vision, and sedation, and the other reported no adverse effects. The total 16 children with drug-resistant epilepsy had a good response to adjunctive LCM therapy without severe adverse effects. The generalizability of the findings from a relatively small sample size, especially the number of patients with a stroke etiology and the pediatric patients with drug-resistant focal epilepsy was probably limited. Additional data would be necessary. Nevertheless, these retrospective observational studies supported the efficacy and safety of LCM in patients with poststroke NCSE.

#### 3.2.3 Case Reports

One case report was included in the literature review. Ylikotila *et al.* [27] reported the first case of LCM use during pregnancy and lactation. It was a case report on cerebral venous thrombosis (CVT) complicated by status epilepticus in early pregnancy. The patient was treated with standard ASMs, including levetiracetam (3000 mg/day) and fosphenytoin (500 mg/day). LCM (300 mg) and lorazepam (6 mg) were added later to address seizures and epileptiform discharges revealed on EEG. After the successful cessation of status epilepticus, levetiracetam and LCM were continued as the only ASMs throughout the rest of the pregnancy. Both levetiracetam and LCM were present in cord blood at levels similar to those in maternal blood. The LCM level in milk was low, resulting in an estimated relative infant dose of 1.8% of the maternal weight-adjusted daily dose in a fully breast-fed infant. The case study demonstrated that LCM is well tolerated and effective with CVT, a relatively smaller stroke subpopulation, worth our attention. In summary, the above clinical studies demonstrated that LCM is well tolerated and effective with CVEE.

### 3.3 Animal Studies with LCM on Poststroke Seizures

Mazzocchetti *et al.* [28] investigated the role of neuroprotection of LCM against *in vitro* ischemia caused by oxygen and glucose deprivation in striatal and hippocampal tissues. They observed that LCM reduced neuronal firing activity in a use-dependent manner without influencing physiological synaptic transmission, which was suitable as an antiepileptic drug. They further observed that LCM could, in a dose-dependent manner, protect neurons in the striatal and hippocampal areas from energy metabolism failure caused by oxygen and glucose deprivation. Ahn *et al.* [29] investigated the neuroprotective effects of LCM against transient cerebral isehcmia-induced neuronal cell damage in the hippocampal cornu ammonis (CA)-1 region by using a gerbil model. They revealed that LCM (25 mg/kg, either presurgical or postsurgical treatment of 5 min ischemia) protected CA1 pyramidal neurons from ischemic injury at 5 days postischemia, evidenced by a reduction of spontaneous motor activity. Additionally, 25 mg/kg LCM markedly attenuated the activation of astrocytes and microglia in the ischemic CA1 region, implying its neuroprotective effects following ischemia. These animal experiments demonstrated that in addition to the anticonvulsant effect, LCM has a neuroprotective effect in animal models of ischemia, in a dose-dependent manner. This study supported the interesting notion that LCM could potentially serve as a neuroprotective agent in patients with ischemic insults, in addition to the treatment of epileptic seizures.

### 3.4 LCM is Relatively Safe in Patients with PSSE when Co-administering Anticoagulants

LCM was generally well tolerated in patients with PSSE. A lack of interaction was observed between LCM and traditional anticoagulants (warfarin) [30]. An increasing number of patients with stroke related to nonvalvular atrial fibrillation are using novel oral anticoagulants (NOACs) for secondary stroke prevention [31]; therefore, it is critical to know whether LCM can be safely coadministered with NOACs. NOACs, including dabigatran, apixaban, rivaroxaban, and edoxaban, act as substrates for permeability glycoprotein (P-gp), an efflux transporter located in the gastrointestinal lumen. Moreover, apixaban, rivaroxaban, and edoxaban are substrates of the cytochrome P-450 system, particularly the CYP3A4 isoform [32]. ASMs that inhibit CYP3A4 or P-gp activity may raise NOAC levels and increase the risk of bleeding; conversely, ASMs that induce CYP3A4 or P-gp activity may reduce NOAC levels and increase antithrombotic efficacy [33]. The 2018 European Heart Rhythm Association Practical Guide advises against the use of carbamazepine, levetiracetam, phenobarbital, phenytoin, topiramate, and valproic acid due to potential drug-drug interactions [34]. Previous observational studies have also demonstrated that concomitant use of NOACs and ASMs was associated with higher risks of thromboembolic and major bleeding events [33, 35-38]. Notably, LCM has exhibited low potential for drug-drug interactions, including with NOACs [34, 39]. LCM acted as neither an inducer nor an inhibitor of CYP3A4 and P-gp in *in vitro* studies; thus, it might be a proper treatment choice for patients with PSSE [33, 38]. Although a few instances of cardiac arrhythmia were associated with LCM [40, 41], most of the clinical studies of LCM verified its general safety and tolerability at clinically recommended doses [21, 42, 43].

## CONCLUSION

This literature review demonstrates LCM’s therapeutic potential for patients with PSSE. The experimental studies on animal models of ischemia revealed favorable antiseizure and neuroprotective effects of LCM. The clinical prospective, retrospective, and case studies in the literature demonstrated and supported the role of LCM as an effective ASM with relatively high safety and tolerability in patients with PSSE, including in patients with poststroke refractory status epilepticus and pregnancy complicated by stroke.

## Figures and Tables

**Fig. (1):**
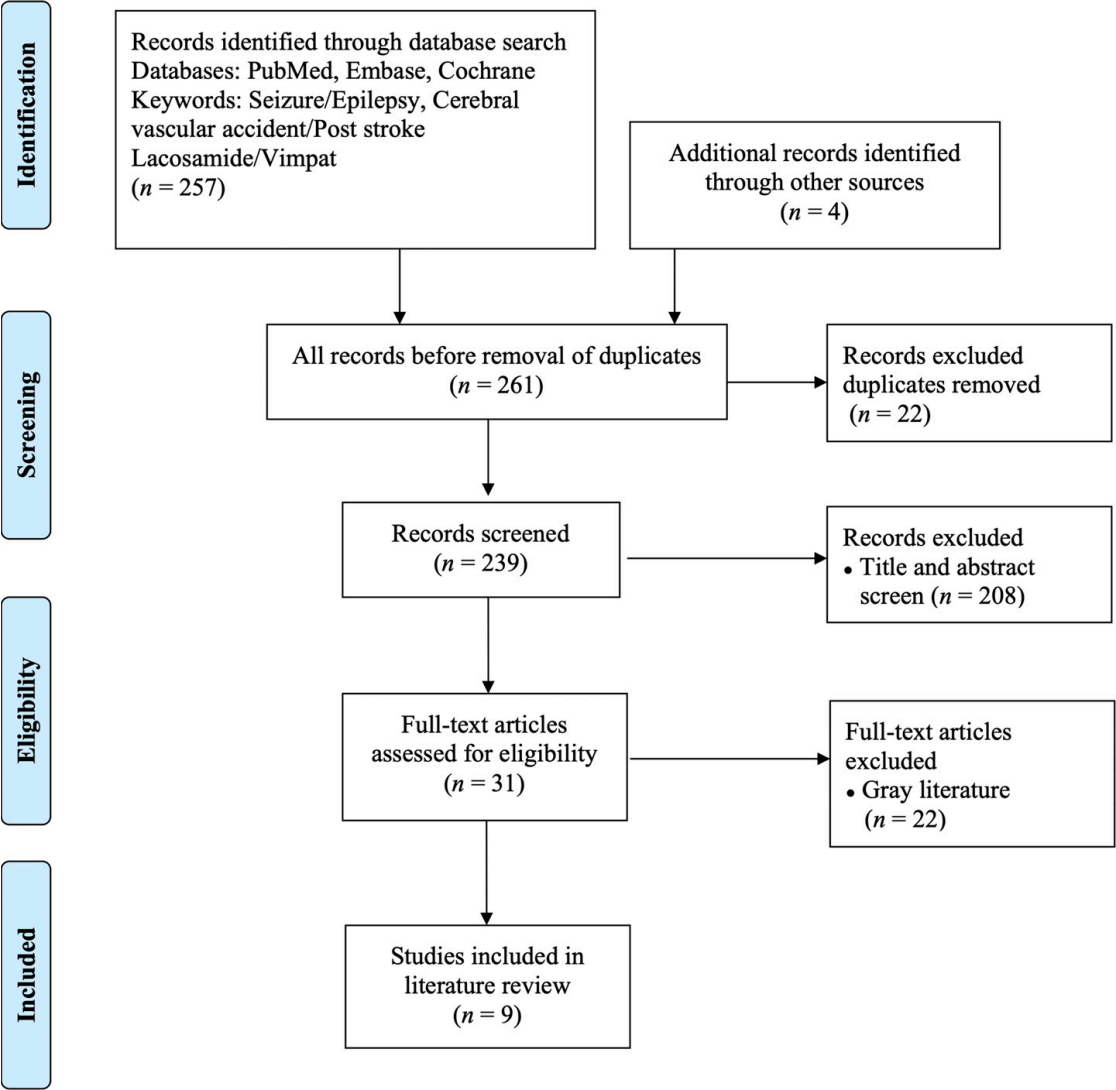
PRISMA flowchart.

**Table 1:** Characteristics of studies included in the literature review.

**Author/Year**	**Purposes/Design**	**Sample Size/** **Population**	**Interventions (per Day)/** **Comparison**	**Main Results**
Rosenow *et al.,* 2020	Tolerability and efficacy/Prospective study	174/Adults with CVEE Monotherapy trial: 61 Conversion group: 30 Observational study: 83	LCM: 100-600 mg/ CBZ-CR: 200-1200 mg LCM: 200-400 mg/Nil Based on physician discretion/Nil	1. LCM monotherapy suggesting numerically better efficacy than CR. 2. Lacosamide was generally well tolerated and effective in patients with CVEE.
Sharma *et al.,* 2019	Effectiveness and EEG/Prospective study	52/RSE (Most common etiology as ischemic stroke)	200 mg (intravenous)/Nil	3. No significant change to renal function after LCM treatment. 4. Response to LCM: 17.3-78.8% (post LCM 4-48 h)
Belcastro *et al.,* 2013	Efficacy and safety/ Retrospective study	16/Post stroke with NCSE	LCM 400 mg/Nil	5. Epileptic activity disappeared in nearly 50% of patients within 45-60 min with no adverse events.
Mnatsakanyan *et al.,* 2012	Efficacy for refractory NCSE/Retrospective study	10/NCSE (1 with chronic infarct)	A median loading dose 200-300 mg infusion within 30 min and the maintenance dosage between 100-200 mg per 12 h/Nil	6. More than half resolved (7/10), one with partial response, and two were resistant to therapy.
Guilhoto *et al.,* 2011	Seizure frequency/ Retrospective study	16/Focal epilepsy (2 with infarct)	Median dose 275 mg/Nil	7. Adjunctive therapy with LCM has a beneficial response in drug-resistant focal epilepsy. 8. The two cases with infarct did not show a remarkable reduction in very high baseline seizure frequency.
Ylikotila *et al.,* 2015	Efficacy during pregnancy/ Case report	1 CVT with SE	LCM 300 mg/Nil	9. Levetiracetam and LCM were continued as the only ASMs throughout the rest of the pregnancy. 10. LCM throughout pregnancy and drug level in breast milk were low, and the concentration in the blood of the breast-fed infant 8 d after delivery was approximately 5% of the cord blood level.
Ahn *et al.,* 2015	Efficacy of neuroprotective/ Animals experimentation	42/*Mongolian gerbil (Induction of transient cerebral ischemia)*	LCM 10-25 mg/kg	11. Preischemic and postischemic treatment with LCM 25 mg/kg markedly protected the CA1 region.
Mazzocchetti *et al.,* 2018	Efficacy of neuroprotective/ Animals experimentation	*In vitro* ischemia (oxygen and glucose deprivation)	LCM 200 μM	12. LCM reduced neuronal firing activity in a use-dependent manner. 13. LCM protected neurons from metabolism failure produced by oxygen and glucose deprivation.

**Abbreviations:** Af (atrial fibrillation); AF (atrial flutter); CVEE (cerebral vascular epilepsy etiology); CVT (cerebral venous thrombosis); EEG (*electroencephalography*); LCM (lacosamide); NCSE (nonconvulsive status epilepticus); PACS (partial anterior circulation syndrome); RNSE (refractory nonconvulsive status epilepticus); RSE (refractory status epilepticus); SE (status epilepticus); TACS (total anterior circulation syndrome).

## LIST OF ABBREVIATIONS

ASMs: Antiseizure Medications
CBZ-CR: Carbamazepine-controlled Release
CVEE: Cerebrovascular Epilepsy Etiology
CVT: Cerebral Venous Thrombosis
EEG: Electroencephalography
IV: Intravenous
LCM: Lacosamide
NOACs: Novel Oral Anticoagulants
PSSE: Poststroke Seizures and Epilepsy

## SUPPLEMENTARY MATERIAL

PRISMA checklist is available as supplementary material on the publisher’s website along with the published article.
